# Characterization of acute myocardial infarction by magnetic resonance imaging: correlation with enzymatic and angiographic findings

**DOI:** 10.1186/1532-429X-11-S1-P120

**Published:** 2009-01-28

**Authors:** Aloha Meave, Erick Alexanderson, Gabriela Melendez, Ana L Mendizabal

**Affiliations:** Instituto Nacional de Cardiologia Ignacio Chavez, Mexico, Mexico

**Keywords:** Left Ventricle, Creatine, Acute Coronary Syndrome, Acute Myocardial Infarction, Ischemic Myocardium

## Introduction

Despite successful recanalization of the infarct-related artery, perfusion of the ischemic myocardium is not or is incompletely restored in up to 30% of patients due to microvascular obstruction (MO). The presence of it has been found to be a predictor of adverse events.

CMR allows accurate assessment of function, transmural extent and total size of infarction, and MVO in all segments of the left ventricle.

## Purpose

To explore the relation between enzymatic, angiographic and CMR findings in patients with treated acute myocardial infarction.

## Methods

We included 23 patients with first acute myocardial infarction, all of them were sent to the cath laboratory. Functional imaging, first pass and delayed gadolinium enhancement was performed using a 1.5 T scan (Sonata, Siemens) after the reperfusion treatment. The correlation between enzymatic, CMR (MO, ventricular function) and hemodynamic (TIMI, TMP) findings were evaluated.

## Results

There were 22 males (95%), average age 52 ± 6 years. Fifteen patients had OM. Ejection fraction was slightly better in patients without microvascular obstruction compared with patients with MO (37.55–50.08 vs 31.85–42.7 p = 0.11). There was not correlation between MO with TIMI flow (pre or post procedure) neither TMP. Higher levels of creatine kinase and troponin I were founded in patients with microvascular obstruction, compared with group without MVO. (p = 0.04 and 0.02).

## Conclusion

Although it has been believed that TMP could be a good method to detect microvascular obstructions in patients with acute coronary syndromes, we don't found correlation between hymodinamics and resonance findings. There is correlation between enzymatic and the presence of OM by CMRI, which is in accordance with more myocardial damage.

